# Bidirectional longitudinal study of type 2 diabetes and depression symptoms in black and white church going adults

**DOI:** 10.1186/s40200-015-0150-5

**Published:** 2015-04-14

**Authors:** Octaviana Hemmy Asamsama, Jerry W Lee, Kelly R Morton, Serena Tonstad

**Affiliations:** Department of Psychology, Loma Linda University, 11130 Anderson Street, Central Building, Suite 106, 9235 Loma Linda, CA USA; School of Public Health, Loma Linda University, Loma Linda, CA USA; Department of Family Medicine, Loma Linda University, Loma Linda, CA USA; Department of Endocrinology, Morbid Obesity and Preventive Medicine, Oslo University Hospital, Oslo, Norway

**Keywords:** Type 2 diabetes, Depression, Bidirectional, Black, Adventist

## Abstract

**Background:**

There is a need to longitudinally examine depression and DM2 relationship in a population that values positive health behaviors. The aim of this study was to prospectively investigate the bidirectional relationship between depression and DM2.

**Methods:**

A cohort sample of 4,746 Black (28.4%) and White (71.6%) Seventh-day Adventist adults who participated in the Biopsychosocial Religion and Health Study (BRHS) completed a short form of the Center for Epidemiologic Studies Depression Scale (CES-D) 11 along with self-report of lifetime physician diagnosis of type 2 diabetes (DM2) and treatment of DM2 and/or depression in the last 12 months in 2006–7 and 2010–11. Hierarchical logistic regression analyses were completed to predict risk for future disease while controlling for demographic and health related variables.

**Results:**

While there were no direct effects of depression on later DM2, there was an indirect effect mediated by BMI (effect = 0.13; 95% CIs [0.08, 0.20]) even after controlling for demographic variables as covariates using Hayes’ PROCESS macro mediation analysis. Similarly, there was also only an indirect effect of DM2 on later depression mediated by BMI (effect = 0.13; 95% CIs [0.05, 0.22]) after controlling for demographic variables.

**Conclusions:**

The results highlight BMI as a risk factor for both DM2 and depression. The negative consequences of having higher BMI in conjunction at baseline with another disease can increase the risk for other chronic disease even in a span of 2.04 – 5.74 years, the length of study interval.

The relationship between depression and type 2 diabetes (DM2) has been observed for some time [1]. In adults with DM2, depression is twice as prevalent compared to non-diabetic adults [2]. Meta-analytic studies also determined that risk for diabetes in depressed individuals is up to 60% higher than for those without [3-5]. In contrast, Mezuk et al. [5] reported only a modest increase in the risk of developing depression for individuals with DM2.

There are several longitudinal, bidirectional depression and DM2 studies [6,7]. Palinkas et al. [6] followed a cohort of 971 White adults ages 50 and older for eight years as part of the Rancho Bernardo Heart and Chronic Disease Study. Individuals with current depressive symptoms are at twice the risk of DM2 after controls. Golden et al. [7] followed a cohort of 6,814 individuals as part of a longitudinal study of cardiovascular disease from 2000 to 2005. Risk for new incident DM2 was 1.10 higher with each five-point increase in Center for Epidemiologic Studies Depression Scale (CES-D) score. However, many prior studies have been limited by cross-sectional analyses [8] or populations burdened by high rates of confounding health behaviors like smoking, alcohol use, and physical inactivity [9,10]. Therefore, there is a need to examine depression and DM2 in a population that values positive health behaviors. Seventh-day Adventists have been found to have relatively low rates of confounding health risk factors like smoking and chronic alcohol use [11].

In addition, we wanted to assess the effects of obesity since it is a risk factor for both depression and DM2 [12,13]. Silva, Atlantis, and Ismail [14] reviewed the relationship between depression and insulin resistance in the context of obesity. Depression can precipitate behavioral changes that increase the risk for obesity. Behaviors associated with depression include physical inactivity, excessive intake of high caloric beverages, and smoking cigarettes. Like many other chronic diseases, type 2 diabetes is also highly influenced by environmental factors like diet and exercise. As individuals increase the intake of energy dense foods, there is an elevation of glucose levels. Chronic elevation of glucose levels can impact insulin sensitivity and impair the ability of the pancreas to regulate insulin. Insulin resistance and pancreatic beta-cells dysfunction are biological changes associated with type 2 diabetes [15].

Therefore, the present investigation examined whether baseline depression symptoms (and no DM2) predicted new incident DM2 in a population of older Adventist adults. In addition, we then evaluated whether self-reported DM2 (and no depression symptoms) at baseline predicted later depression symptoms. Finally, several a priori analyses were conducted to examine potential mediators (physical activity, length of study interval, and body mass index).

## Methods

### Participants and procedures

The Biopsychosocial Religion and Health Study (BRHS) is a longitudinal cohort study of Adventist adults in 2006–7 and again in 2010–11. Twenty thousand were randomly sampled from 97,000 US and Canadian participants from the Adventist Health Study-2 (AHS-2) in 2004–6 [16]. The BRHS was developed to better understand the influence of religion on health outcomes [13] via two 20-page questionnaires. The recruitment process included sending the religion and health questionnaire with an initial letter and subsequent reminder cards [16,17]. Participants provided written consent and Loma Linda University’s Institutional Review Board approved the study.

In 2006–7, 10,988 participants completed the self-report BRHS questionnaires and 6,508 completed a similar questionnaire again in 2010–11. For this study, subjects were included if they: (a) participated in both data collections, (b) were Black (African American, Caribbean Black, biracial) or White, (c) were Seventh-day Adventist, (d) had no history of smoking regularly, and (e), completed all self-report measures of depression and DM2.

### Measures

All baseline measures were determined in 2006–7. Measures of depression symptoms and DM2 were also reported in 2010–11. All measures were based on self-report.

### Smoking history

Participants answered the following question: “*have you ever smoked regularly?*” Options included: *no*; *yes, cigars*; *yes, pipe*; *yes, cigarettes*. Individuals were included if they answered “no” to this item.

### Depressive symptoms

Participants completed the CESD-11, a measure of current depressive symptoms in the past week [18]. This abbreviated form has been found to be an accurate and reliable measure of depressive symptoms. For our study, we followed Kohout’s conversion transformation as follows: values were transformed to *z* – scores, then *z* – scores were multiplied by the National Health and Nutrition Examination Survey (NHANES-I) standard deviation; NHANES-I standardized gender specific means were then added to each score. The NHANES-I distributions were standardized norms by gender. The average CES-D scores for men were 7.1 (*SD* = 7.2) compared to 10.0 (*SD* = 9.1) for women. Individuals with NHANES-I adjusted CES-D score ≥ 16 were identified as having elevated depressive symptoms for screening, not diagnostic purposes. This has been found to be indicative of mild to moderate depression [19].

Individuals were categorized as having depression (*yes* or *no*) if they have at least one of the following: CESD score ≥ 16, treatment by a physician for depression in the past 12 months (*yes* or *no*). Incident depression was defined among participants who did not have depression in 2006–7 but developed later depression in 2010–11.

### Diabetes status

Study participants were asked whether they were “*ever diagnosed with diabetes mellitus (type II adult onset) by a physician.*” They also reported whether or not they were ever treated by a physician for DM2 in the past 12 months (*yes* or *no*). In a validation study of self-reported DM2 in a population of Adventist adults, self-report was found to be a relatively valid method for assessing DM2 with sensitivity ranging from 65.2% to 80.5% and specificity ranging from 95.2% to 97.9% depending on diagnostic reference criteria [20]. Participants who answered *yes* to either question were considered diabetic. Incident diabetes was defined among participants who did not have diabetes in 2006–7 but developed DM2 at 2010–11.

### Sociodemographic characteristics

Variables included age (years); gender; ethnicity (*White, Black*); marital status (*never married*, *married, separated, divorced,* and *widowed*). Martial status was then categorized to either *married* or not *married*. Educational attainment was originally a nine-category scale (*grade school, some high school, high school diploma, trade school diploma, some college, associate degree, bachelors degree, masters degree, doctoral degree*). It was aggregated into four categories: high school or less (*grade school, some high school, high school diploma*), some college or associate degree (*trade school diploma, some college, associate degree*), bachelor’s degree, and graduate degree (*masters degree, doctoral degree*). Socioeconomic status was defined by participant’s answer in 2006–7 to “*difficulty meeting family expenses for basic needs in last year*” (*not at all, a little, somewhat, fairly,* and *very*). Participants who reported *a little, somewhat, fairly,* and *very* were then categorized as having low socioeconomic status; *not at all* was then categorized as having high socioeconomic status.

### Length of study interval

The length between BHRS study periods (2006–7 and 2010–11) was measured in days.

### Health behavior covariates

BMI was calculated as self-reported weight (kg)/height (m)^2^. Physical activity was determined by reported frequency, intensity, and duration of vigorous physical activities, such as brisk walking, jogging, and bicycling per week. An activity with vigorous intensity was defined as an activity that “*worked up a sweat, get your heart thumping, or get out of breath*.” The total physical activity value in minutes per week was determined by multiplying the frequency of sessions with the duration of activity. The physical activity questions in this questionnaire have been shown to be both reliable and valid [21,22].

### Statistical analysis

Comparisons between demographic and clinical characteristics were assessed using *t*-tests for continuous variables and χ^2^ tests for categorical variables. Analyses were performed using PASW Statistics Version 20 [23] with *p-*value of <0.05 as the determinant of statistical significance.

A series of hierarchical logistic regression analyses were completed to predict new disease incidence while controlling for other demographic and health variables. The first set of analyses determined whether baseline (2006–7) depression predicted new incident DM2 (2010–11) (*yes, no*). Baseline depression (*yes, no*) was the independent variable with DM2 status (2010–11) as the dependent variable. We first excluded participants with DM2 in 2006–7. The model included the following variables in the listed order: age, gender, ethnicity, education, socioeconomic status, marital status, length of study interval, physical activity, and BMI.

The second set of analyses determined whether participants with self-reported DM2 at baseline (2006–7) were more likely to develop later depression (2010–11) compared to those without (*yes, no*). Baseline 2006–7 DM2 status was the independent variable with follow-up depression in 2010–11 as the dependent variable. Participants with baseline (2006–7) depression were first excluded from the analysis. The model included the following variables in the listed order: age, gender, ethnicity, education, socioeconomic status, marital status, length of study interval, physical activity, and BMI.

Finally, we conducted additional analyses to examine potential mediators using Hayes PROCESS macro [24]. PROCESS is an SPSS add-on that aid in statistical mediation analyses using logistic regression-based models. In addition, it is able to estimate both direct and indirect effects. We controlled for age, gender, ethnicity, education, socioeconomic, and marital status as covariates (order as listed). Length of interval, physical activity, and BMI were evaluated as possible mediators. PROCESS macro coefficients listed are unstandardized.

### Human participation protection

The institutional review board of the Loma Linda University approved this study.

## Results

Of the 6,508 eligible participants who participated in both data collection periods, 1,762 were excluded from analyses. There were significant differences between those who were included versus excluded in all baseline variables except socioeconomic status (see Table 1). The final sample consisted of 1,441 males (30.4%) and 3,305 females (69.6%) with a mean age of 61.3 years (*SD* = 12.7). The majority of the participants were White with some college or higher degrees of education, reported no financial difficulties in the year prior to 2006–7, and were married. The length of interval between the two study collection periods (2006–7 to 2010–11) ranged from 744 days (2.0 years) to 2,095 days (5.7 years). Although the average BMI was 26.2 (*SD* = 5.7), 1,012 participants (21.3%) were obese with a BMI greater than 30.

**Table 1:** **Characteristics of participants and excluded at baseline (2006–2007)**

	**Participants**	**Excluded** ^**a**^	
**Mean (** ***SD*** **)**	***n*** **= 4,746**	***n*** **= 1,762**	***p*** **-value**
Age (years)	61.3 (12.7)	63.7 (12.1)	<0.001
Female (%)	69.6	61.8	<0.001
White (%)	71.6	57.2	<0.001
*Level of education* (%)			<0.001
Trade, high school, or less	13.4	22.8	
Some college or Associate’s degree	34.6	43.2	
Bachelor’s degree	26.8	18.6	
Graduate degree	25.3	15.4	
Low socioeconomic status^b^ (%)	25.8	27.4	0.2
Married (%)	59.1	43.3	<0.001
Length of study interval (days)	1,320.0 (220.0)	1,369.7 (249.3)	<0.001
Vigorous exercise (minutes/week)	84.7 (93.7)	75.0 (91.7)	<0.001
Body mass index (kg/m^2^)	26.6 (5.7)	27.5 (6.2)	<0.001
Depression^c^ (%)	18.6	23.1	<0.001
Type 2 diabetes^d^ (%)	8.5	12.6	<0.001

*Notes.* For continuous variables, *t*-test was used to determine baseline differences. Differences in categorical variables were analyzed using *χ* ^2^ test.

^a^Participants were excluded if they had missing variables for either depression or DM2, reported as *Others* in their ethnicity, history of regular smoking, and/or did not report as an active or inactive Seventh-day Adventist.

^b^Low socioeconomic status was defined as the number of participants who reported *a little, somewhat, fairly,* and *very difficult* meeting expenses for basic needs in the last year.

^c^Participants were identified as depressed if they had CESD score ≥16 and/or reported treatment for depression in the past 12 months in 2006–7.

^d^Type 2 diabetes status referred to participants answering “yes” to either of the following: “*ever diagnosed with diabetes mellitus (Type II adult onset) by a physician?*” and/or “*treated by a physician for diabetes mellitus (Type II adult onset) in the last 12 months*?” in 2006–7.

Ethnicity was examined and did not moderate the associations between diabetes and depression.

### Baseline depression and later DM2

At baseline (2006–7), 18.6% (*n* = 882) were identified as depressed based on CED ≥ 16 and/or reported depression treatment in the past year. Individuals with depression were significantly different in all demographic and health measures compared to individuals without depression except for ethnicity and length of study interval. Consistent with previous literature [5,6], the rates of DM2 almost doubled in depressed participants (13.3%) compared to those without depression (7.5%) at baseline (2006–7).

There were 405 (8.5%) individuals excluded due to baseline DM2. Table 2 summarizes demographic, lifestyle, and rates of DM2 in 2006–7 by depression status for individuals without DM2 in 2006–7. Baseline depression status (2006–7) was associated with new incidents of DM2 (2010–11), χ^2^ (1, *N* = 4,341) = 6.50, *p* = 0.01 with higher rates of later DM2 (3.8%) for individuals with depression versus those without depression (2.2%). After adjusting for age, gender, ethnicity, education, socioeconomic and marital status, the association of depression with later (2010–11) DM2 was not statistically significant, OR = 1.01, 95% CI, [0.27 – 3.79], *p* = 0.99. The relationship continued to be non-significant after length of study interval, physical activity, and BMI were added (see Table 3). In addition, the interaction between ethnicity and depression status in 2006–7 was not a significant predictor of DM2 at follow up (2010–11).

**Table 2:** **Baseline (2006–2007) characteristics of individuals with and without depression**

	**Not depressed**	**Depressed** ^**a**^	
**Mean (** ***SD*** **)**	***n*** **= 3,576**	***n*** **= 765**	***p*** **-value**
Age (years)	61.09 (12.79)	59.31 (12.77)	<0.001
Female (%)	66.8	83.9	<0.001
White (%)	71.8	75.4	0.04
*Level of education* (%)			<0.001
Trade, high school, or less	12.9	14.9	
Some college or Associate’s degree	32.8	40.7	
Bachelor’s degree	27.4	26.3	
Graduate degree	26.9	18.1	
Low socioeconomic status^b^ (%)	22.7	38.3	<0.001
Married (%)	61.1	53.8	<0.001
Length of study interval (days)	1320.65 (220.42)	1322.41 (225.06)	0.84
Vigorous exercise (minutes/week)	91.62 (95.49)	62.86 (82.98)	<0.001
Body mass index (kg/m^2^)	25.96 (5.09)	27.31 (6.30)	<0.001
New incident of type 2 diabetes^c^ (%)	2.2	3.8	0.01

*Notes.* For continuous variables, *t*-test was used to determine baseline differences. Differences in categorical variables were analyzed using [2] test.

^a^Participants were identified as depressed if they had CESD score ≥16 and/or reported treatment for depression in the past 12 months in 2006–7.

^b^Low socioeconomic status was defined as the number of participants who reported *a little, somewhat, fairly,* and *very difficult* meeting expenses for basic needs in the last year.

^c^New incident of type 2 diabetes status referred to participants answering “yes” to either of the following: “*ever diagnosed with diabetes mellitus (Type II adult onset) by a physician?*” and/or “*treated by a physician for diabetes mellitus (Type II adult onset) in the last 12 months*?” in 2010–11 but answered “no” to either questions in 2006–7.

**Table 3:** **Hierarchical logistic regression predicting new incidents of type 2 diabetes**

	**95% Confidence interval**			
***Demographic***	**OR**	**Lower**	**Upper**	***p*** **-value**
Age (years)	1.04	1.02	1.06	<0.001
Gender	0.75	0.44	1.28	0.30
Ethnicity	0.85	0.23	3.09	0.80
Education level	1.16	0.93	1.43	0.18
Socioeconomic status	1.16	0.73	1.85	0.52
Marital status	1.28	0.83	1.96	0.27
Length of study interval (days)	1.00	1.00	1.00	<0.001
Vigorous exercise (min/week)	1.00	1.00	1.00	0.63
Body mass index (kg/m^2^)	1.13	1.10	1.16	<0.001
Depression^a^ (2006–7)	0.77	0.19	3.12	0.71
Depression X Ethnicity	1.70	0.65	4.41	0.28

*Notes.* ^a^Participants were identified as depressed if they had CESD score ≥16 and/or reported treatment for depression in the past 12 months in 2006–7.

We conducted additional analyses to examine potential mediators using Hayes PROCESS macro [24]. We also controlled for age, gender, ethnicity, education, socioeconomic, and maritalstatus as covariates in the PROCESS analysis. While there were no direct effects of depression on later DM2 (effect = 0.45; 95% CIs [−0.03, 0.93]), there was an indirect effect of depression on later DM2 (2010–11) mediated by BMI (effect = 0.13; 95% CIs [0.08, 0.20]). Depression was positively related to BMI and higher BMI increased the risk of DM2. Of note, length of clinic visit and physical activity were not significant mediators of depression and later DM2 (effect = 0.002; 95% CIs [−0.03, 0.03]), (effect = −0.02; 95% CIs [−0.08, 0.05]) respectively.

### Baseline DM2 and later depression

There were no significant differences at baseline (2006–7) between the groups by gender, socioeconomic status, or length of study interval. The rate of depression at baseline (2006–7) was higher for those with DM2 (28.9%) compared to those without DM2 (17.6%). There were 882 (18.58%) of participants who were excluded due to baseline depression status. Table 4 lists demographic and health measures by DM2 status for individuals without depression in 2006–7.

**Table 4:** **Baseline (2006–2007) characteristics of individuals with and without type 2 diabetes**

	**Not type 2 diabetes**	**Type 2 diabetes** ^**a**^	
**Mean (** ***SD*** **)**	***n*** **= 3,864**	***n*** **= 288**	***p*** **-value**
Age (years)	61.09 (12.79)	67.48 (10.18)	<0.001
Female (%)	66.8	62.8	0.18
White (%)	71.8	61.1	<0.001
*Level of education* (%)			0.27
Trade, high school, or less	12.9	13.6	
Some college or Associate’s degree	32.8	36.6	
Bachelor’s degree	27.4	22.3	
Graduate degree	26.9	27.5	
Low socioeconomic status^b^ (%)	22.7	20.7	0.43
Married (%)	61.1	54.7	0.03
Length of study interval (days)	1,320.65 (220.42)	1,305.49 (205.92)	0.26
Vigorous exercise (minutes/week)	91.62 (95.49)	73.19 (91.06)	<0.01
Body mass index (kg/m^2^)	25.96 (5.09)	30.16 (6.42)	<0.001
New incident of depression^c^ (%)	8.8	12.2	0.06

*Notes.* For continuous variables, *t*-test was used to determine baseline differences. Differences in categorical variables were analyzed using [2] test.

^a^Self-reported DM2 was defined as “yes” if participants answered “yes” to “*ever diagnosed with diabetes mellitus (Type II adult onset) by a physician?*” and/or reported treatment for type 2 diabetes in the last 12 months in 2006–7.

^b^Low socioeconomic status was defined as the number of participants who reported *somewhat, fairly,* and *very difficult* meeting expenses for basic needs in the last year.

^c^Participants were identified as depressed if they had CESD score ≥16 and/or reported treatment for depression in the past 12 months in 2010–11 but answered “no” to treatment and had CESD score <16 in 2006–7.

DM2 status at baseline (2006–7) was not statistically significant with new incidents of later depression (2010–11) after controlling for demographic information, OR = 1.31, 95% CI, [0.42– 4.13], *p* = 0.64. The relationship between DM2 (2006–7) and later depression (2010–11) remained non-significant after adding the length of study interval, physical activity, and BMI to the model (see Table 5). The interaction between ethnicity and DM2 in 2006–7 was not a significant predictor of new incidence of later depression at follow up (2010–11).

**Table 5:** **Hierarchical logistic regression predicting new incidents of depression**

	**95% Confidence interval**			
***Demographic***	**OR**	**Lower**	**Upper**	***p*** **-value**
Age (years)	1.00	0.99	1.01	0.93
Gender	0.71	0.54	0.92	0.01
Ethnicity	0.58	0.22	1.52	0.27
Education level	0.93	0.82	1.05	0.22
Socioeconomic status	1.42	1.10	1.83	0.01
Marital status	1.38	1.09	1.75	0.01
Length of study interval (days)	1.00	1.00	1.00	0.14
Vigorous exercise (min/week)	1.00	1.00	1.00	0.19
Body mass index (kg/m^2^)	1.03	1.01	1.05	<0.001
Diabetes^a^ (2006–7)	1.11	0.35	3.54	0.86
Diabetes X Ethnicity	1.10	0.48	2.51	0.82

*Notes.* ^a^Self-reported DM2 was defined as “yes” if participants answered “yes” to “*ever diagnosed with diabetes mellitus (Type II adult onset) by a physician?*” and/or reported treatment for type 2 diabetes in the last 12 months in 2006–7.

Again, we examined the potential mediators using Hayes PROCESS macro while controlling for age, gender, ethnicity, education, socioeconomic, and marital status as covariates. There was no direct effect of DM2 on later depression (effect = 0.23; 95% CIs [−0.16, 0.63]). However, there was an indirect effect mediated by BMI (effect = 0.13; 95% CIs [0.05, 0.22]). DM2 was positively associated with BMI and higher BMI increased the risk for later DM2. Of note, length of study interval and physical activity were not significant mediators of the DM2 and depression relationship (effect = − 0.002; 95% CIs [−0.02, 0.01]), (effect = 0.01; 95% CIs [−0.003, 0.05]) respectively.

## Discussion

This prospective study of community dwelling Adventist adults investigated the bidirectional relationship between depression symptoms and DM2. It would appear that there was no direct bidirectional relationship between depression and DM2. This is consistent with the literature [14] where differences were noted only in cross-sectional studies but not prospective studies. However, this relationship was mediated by an indirect effect of BMI. In both direction of depression symptoms and DM2, the baseline disease increased the risk for having higher BMI at baseline, which in turn increased the risk for the other disease process. In addition, BMI continued to be a significant mediator even after controlling for demographic variables, the duration between clinic visits, and physical activity.

Of note, there are baseline socioeconomic differences unique to individuals with elevated the depression symptoms to later DM2 sample population. Notably, there are higher rates of females (83.9% versus 62.8%), individuals with less than graduate level degree (81.9% versus 72.5%), and experienced difficulty meeting expenses in the past year (38.3% versus 20.7%). A large population study found that chronic economic hardship have cumulative health effects on health, including high levels of depressive symptoms, pessimism, and hostility [25]. They found that chronic economic hardship placed individuals at a higher risk for clinical depression compared to type 2 diabetes. Perhaps a similar dose–response association can explain the differences in our sample population.

Other studies have examined the depression and diabetes relationship. For example, Palinkas et al. [6] followed a cohort of 971 adults ages 50 and older for eight years as part of the Rancho Bernardo Heart and Chronic Disease Study. They found that Beck Depression Inventory (BDI) score ≥ 11 doubled the risk for DM2 after controls. There was no significant evidence that showed DM2 was a predictor of a positive depression screen though new depression incidence was low in this study. However, since they only adjusted for BMI and it is unknown whether or not BMI was a moderator for this study. Finally, they have a longer study duration, which might explain the significant depression to new incident of DM2 relationship. We hypothesized that a significant depression to DM2 relationship might emerge later if differences in chronic economic hardship persist in the sample population with elevated depressive symptoms. However, since chronic economic hardship was not a study variable, caution should be exercised in interpreting the cause and possible outcome of baseline socioeconomic vulnerabilities in participants with elevated depressive symptoms.

Given the persistent effect of BMI on the depression and DM2 relationship, it should be extrapolated that reducing the indirect effect of BMI on depression and DM2 is the main implication of our findings. Perhaps integrating treatments that focused on individuals with greater risk would help reduce this indirect effect. For example, depressed individuals who are experiencing weight gain might benefit from behavioral interventions focusing on weight loss or anti-inflammatory medications in order to reduce the risk for later DM2. The negative consequences of having higher BMI in conjunction at baseline with another disease can increase the risk for other chronic disease even in a span of 2.04 – 5.74 years.

Our results should be considered in light of the following strengths and weakness in the study design. The strength of the study lies in the prospective analysis of the relationship between depression and DM2 in a large population of older adults. There is also a large representative sample of Black participants. Future studies could also examine the differences in later DM2 for individuals whose depression persisted over time compared to those whose depression improved over time.

Limitations include the potential generalizability of the findings given the study population. Adventists might not accurate depict the general population experiencing depression and type 2 diabetes. In older age groups the BRHS sample has better reported physical and mental health compared to the national norms for the same age groups [17]. Also, our population has been found to have better glycemic control [20] compared to the US population [26]. Perhaps this could be attributed to Adventist’s religiously based health behavior recommendations such as regular exercise, healthy diet, and abstaining from smoking or alcohol consumption [11]. However, utilizing this population helped reduced potential confounding factors such as smoking or history of regular drinking. Finally, since the study population was healthier with lower BMI compared to the excluded participants, it is possible that the true strength of findings might have underestimated.

The results from this study have implications to the importance of additional support for depression screening and intervention for individuals with DM2. Given that depression could be risk factor for later DM2, healthcare practitioners should be increasingly aware of relationship. Patients might benefit from additional psychoeducation about said relationship and providers should also screen their diabetic clients for depression and, if appropriate, encourage them to seek additional care for psychological distress like depression. Addressing the depression and diabetes relationship more aggressively could have bigger implications in overall health policy and cost since there is a significantly higher cost for diabetes patients with depression versus those without depression [27]. It is especially important to provide treatment for those with higher BMI since it would appear that BMI is the link between these two chronic diseases.

## Abbreviations

AHS: Adventist health study
BRHS: Biopsychosocial religion and health study
CES-D: Center for epidemiologic studies depression scale
DM2: type 2 diabetes
NHAES: National Health and Nutrition Examination Survey
